# Impact on immigrant screening adherence with introduction of a population‐based colon screening program in Ontario, Canada

**DOI:** 10.1002/cam4.2026

**Published:** 2019-02-21

**Authors:** Amina Moustaqim‐Barrette, John J. Spinelli, Arminée Kazanjian, Trevor J.B. Dummer

**Affiliations:** ^1^ School of Population and Public Health University of British Columbia Vancouver British Columbia Canada; ^2^ Population Oncology BC Cancer Vancouver British Columbia Canada

**Keywords:** cancer screening, colon cancer, colorectal cancer, equity, immigrant health, program evaluation

## Abstract

**Introduction:**

The literature suggests that differential colorectal cancer (CRC) screening adherence exists between Canada’s immigrant and nonimmigrant populations. This study explores the impact of Ontario’s population screening program, *ColonCancerCheck*, on CRC screening uptake in immigrant and nonimmigrant population groups.

**Methods:**

Data from 2005, 2007‐2008, and 2011‐2012 was obtained from the Canadian Community Health Survey, to represent the intervention periods (the time periods before, during, and after implementation of the *ColonCancerCheck *intervention). Multivariable logistic regression was used to examine the effect of immigration status on the risk of nonadherence to guideline‐recommended CRC screening, and an interaction analysis was performed to determine whether the screening differential between immigrant and nonimmigrant populations changed upon introduction of the *ColonCancerCheck *program.

**Results:**

Recent and long‐term immigrants were both at increased risk of CRC screening nonadherence compared to the Canadian‐born population (OR 3.73 (CI 2.25‐6.18) and OR 1.24 (CI 1.13‐1.36), respectively). While not statistically significant, there was an attenuation of the risk of nonadherence to screening for recent immigrants compared with Canadian‐born individuals after the implementation of the *ColonCancerCheck* program.

**Conclusions:**

This study provides evidence of a screening differential between immigrants and nonimmigrants, and suggests that the implementation of the *ColonCancerCheck* screening program in Ontario may have increased colon screening uptake amongst recent immigrants. Further studies are needed to address the factors leading to inequities in immigrant CRC screening adherence.

## 1 INTRODUCTION

Colorectal cancer (CRC) is the second most commonly diagnosed cancer in Canada, as well as the second leading cause of cancer death in men and third leading cause of cancer death in women.^1^ Importantly, the reliability and efficacy of CRC screening is well established, with several randomized controlled trials (RCTs) demonstrating 15%–33% reductions in CRC mortality as a result of screening with Fecal Occult Blood Tests (FOBT) or sigmoidoscopy.^2^, ^3^ However, the literature also indicates that a differential exists in CRC screening uptake between Canada's immigrant and nonimmigrant populations.^4^, ^5^ In 2009, CRC screening rates were 41% among immigrants, compared with 55% in the nonimmigrant population.^6^ With projected increases in the Canadian immigrant population from 20% in 2006 to 25% to 28% by 2031,^7^ it is important to monitor immigrant health and healthcare access in Canada, including preventative screening care, to reduce avoidable inequities. Importantly, the province of Ontario is Canada's most populous province – home to more than 13 million of the country's 35 million people and to more than half of all new immigrants to Canada.^8^

In 2001, the Canadian Task Force on Preventive Health Care (CTHPHC) published the first recommendations for CRC screening; FOBT every 2 years or sigmoidoscopy every 10 years.^9^ In 2007, the province of Ontario announced its intentions to implement a province‐wide CRC screening program, *ColonCancerCheck*, which was rolled out between 2007 and 2008. By the end of 2011, programmatic CRC screening and mail‐out invitations had been made available to 100% of screening‐eligible individuals in Ontario.^10^ Prior to the introduction of the *ColonCancerCheck *program, screening for colorectal cancer in Ontario using FOBT or sigmoidoscopy was accomplished through opportunistic screening of at‐risk individuals. This relied on physician recommendation or patients raising the issue during appointments or regular checkups, which led to very low screening rates among asymptomatic individuals.^11^ The *ColonCancerCheck *program involves mailout invitations to average risk individuals aged 50−74 to complete a gFOBT test at their primary care physician (PCP) office once every 2 years, and instructions and distribution of standardized gFOBT kits to physician offices, pharmacies, and hospitals in Ontario. Provisions are also made for individuals with no PCP.^12^

While some studies have shown improvements to screening rates in the general population as a result of CRC screening programs – including a 2013 study that found that the *ColonCancerCheck* program increased FOBT screening in the average risk population by 5.2%^13^ – it is not yet known whether programmatic screening leads to increased screening uptake of immigrants. Other studies evaluating breast and cervical cancer screening rates have found that despite the existence of programmatic screening in Canada, immigrant‐related inequities in screening participation persist.^14^, ^15^, ^16^, ^17^

This study uses multiple yearly cycles of a pan‐Canadian health survey to compare rates of CRC screening among immigrants and nonimmigrants in the period prior to, during, and after complete implementation of the *ColonCancerCheck* population screening program, in order to determine whether the implementation of the program was associated with increased screening participation. Results from this study provide a better understanding of the effect of population screening programs on the uptake of immigrant populations as compared to Canadian‐born individuals, and will inform improvements and revisions to screening programs across Canada.

## 2 MATERIALS AND METHODS

### 2.1 Study design

Data for this study were extracted from the 2005, 2007‐2008, and 2011‐2012 cycles of the Canadian Community Health Survey (CCHS).^18^, ^19^, ^20^ The CCHS is a nationally representative cross‐sectional survey conducted by Statistics Canada. The survey collects self‐reported data on a variety of health status, health determinants, and health service utilization factors of Canadians aged 12 years and older. The CCHS sampling frame represents approximately 98% of the Canadian population, with individuals living on First Nations’ Reserves, in certain remote regions, or who are full‐time members of the Canadian Forces among those excluded from the sampling frame. The survey uses a multistage cluster sampling strategy to conduct over 65,000 in‐person interviews annually with an overall response rate of 79.1% in 2005, 84.6% in 2007‐2008, and 86.9% in 2011‐2012.^18^, ^19^, ^20^ A detailed description of the CCHS's sampling and interviewing methods is available from Statistics Canada.^21^ The CCHS relies on landlines to contact participants and does not guarantee the availability of a translator for interviews, and thus may be susceptible to participation bias.

The dataset used in this study is included in Statistics Canada's public use microdata files (PUMFs), as part of their Data Liberation Initiative (DLI). Study‐specific ethics approval was not required as it was covered by the publicly available data clause (Item 7.10.3) governing the use of public release data set under the University of British Columbia's Policy #89: Research and Other Studies Involving Human Subjects.^22^

### 2.2 Analytic sample

The current analysis combines three cycles of data from the CCHS to: (a) evaluate the screening differential between immigrants and nonimmigrants, and, (b) assess the impact of Ontario's *ColonCancerCheck *population‐based screening program on CRC screening uptake in these population subgroups. The analysis was limited to individuals aged 50‐74 years living in Ontario with valid responses to the CRC screening module, who were not living with cancer, who did not have a family history of CRC, and who had no history of inflammatory bowel disease, including Crohn's and ulcerative colitis, at the time of interview in order to capture average‐risk individuals.

**Figure 1:**
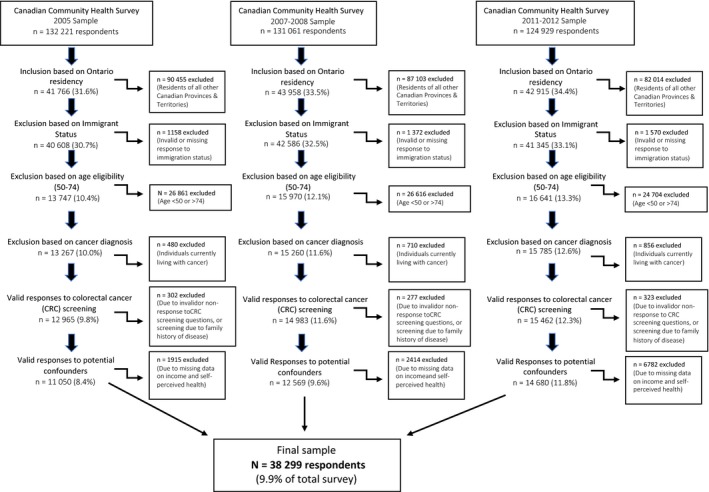
Study sample from CCHS 2005, 2007‐2008, and 2011‐2012 for analysis of relationship between colorectal cancer screening program implementation and screening uptake among immigrants in Ontario

Of the combined 388 211 respondents (n = 132 221 in 2005, n = 131 061 in 2007‐2008, and n = 124 929 in 2011‐2012), approximately 33% (n = 128 639) of the sample were included for their Ontario residency. From here, 3.2% (n = 4100) were excluded due to invalid answers to immigration status. Of the remaining observations, around 37% (n = 46 358) met the CRC screening age criteria of 50‐74 years. Around 4% (n = 2046) of these were excluded due to reporting living with cancer or having a family history of CRC. Of the remaining 43,410 participants, around 6% (n = 2966) were excluded due to invalid or nonapplicable responses to the CRC screening questions. A final 11.5% of the remaining sample (n = 4899) were excluded due to having inflammatory bowel disease or for invalid responses to confounders associated with household income, educational attainment, and self‐perceived health. The final analytic sample included 38 299 respondents that met the inclusion criteria and provided valid responses to the study variables. For detailed methods of sample selection in separate survey cycles, see Figure 1.

### 2.3 Study variables

The primary outcome in this study was CRC screening nonadherence. This binary outcome variable indicated screening was “yes” if the respondent had a FOBT within the last 2 years or endoscopy within the last 10 years, and “no” if they had not had any screening or screening outside the *ColonCancerCheck *time guidelines. In the CCHS, FOBT is described for respondents as a “test to check for blood in your stool, where you have a bowel movement and use a stick to smear a small sample on a special card”. This represents the procedure for both Guaiac and immunochemical‐based FOBTs. Endoscopy is described for respondents as “a colonoscopy or sigmoidoscopy is when a tube is inserted into the rectum to view the bowel for early signs of cancer and other health problems.”^23^ Studies have suggested that self‐reporting CRC screening use is fairly accurate and shows good concordance with administrative and medical record data.^24^

The primary independent variable in the study was immigration status (three categories) where “Recent Immigrants” describes those who arrived in Canada within 0‐9 years before the survey year, “Long‐term Immigrants” describes those who arrived in Canada 10 or more years before the survey year, and “Canadian‐born individuals” describes nonimmigrants. The secondary independent variable was the *ColonCancerCheck* intervention periods (three categories), where 2005 denotes the “pre‐intervention period”, 2007‐2008 the “roll‐out period”, and 2011‐2012 the “post‐intervention period” for the *ColonCancerCheck* program.

Potential confounders included age (5‐year age grouping between 50 and 74), sex (male or female), household income (<$20 000, $20 000‐39 999, $40 000‐59 999, $60 000‐79 999 K and>$80 000 in CAD), educational attainment (less than secondary school, secondary school graduation, some postsecondary, and postsecondary graduation), and self‐perceived health (excellent, very good, good, fair, poor). These covariates were identified as being important predictors of CRC screening behaviors from previous studies and chosen based on data availability.^13^

### 2.4 Analysis

All analyses were conducted using SAS University Edition,^25^ and statistics were weighted using sampling weights provided by Statistics Canada to provide more accurate estimates of variance and to account for uneven probabilities of selection.

Frequency distributions were used to describe differences in study sample characteristics across immigration groups. Multivariable logistic regression model^26^ was utilized to investigate the relationship between immigration status and CRC screening adherence among Ontario residents while adjusting for the confounding effect of intervention period, age, sex, household income, educational attainment, and self‐perceived health. An interaction analysis was conducted in order to determine whether the introduction of the *ColonCancerCheck *program affected CRC screening adherence of differently for immigrant and nonimmigrant populations.

## 3 RESULTS

### 3.1 Descriptive statistics

Table 1 presents the baseline characteristics of 50–74‐year‐old Ontarians, stratified by immigration status. The overall study sample (n = 38 299) was unevenly distributed across immigration groups, with Canadian‐born individuals representing the largest group (76.8%, n = 29 426), long‐term immigrants representing the second largest group (22.4%, n = 8577), and recent immigrants representing 0.77% (n = 296) of the total sample.

**Table 1:** Summary characteristics of Canadian Community Health Survey Respondents (2005, 2007‐08, and 2011‐12), investigation of the association between colorectal cancer screening adherence and *ColonCancerCheck* screening program intervention, stratified by immigration status

Characteristics	Recent immigrants n = 296	Nonrecent immigrants n = 8577	Canadian‐born n = 29 426	Total N = 38 299
	n (%)	n (%)	n (%)	
Lifetime colorectal cancer screening				
Ever	106 (28.8)	5340 (58.6)	18 818 (63.7)	24 264 (39.0)
Never	190 (71.2)	3237 (41.4)	10 608 (36.3)	14 035 (61.0)
Screening adherence				
Yes	86 (22.5)	4534 (50.3)	15 791 (53.9)	20 411 (51.8)
No	210 (77.5)	4043 (49.7)	13 635 (46.1)	17 888 (48.2)
Sex				
Male	137 (54.3)	3898 (49.7)	13 167 (49.2)	17 202 (49.5)
Female	159 (45.7)	4679 (50.3)	16 259 (50.8)	21 097 (50.5)
Time period				
Preintervention (2005)	84 (28.5)	2580 (28.5)	8386 (28.7)	11 050 (28.7)
Roll‐out (2007‐08)	116 (32.5)	2924 (32.4)	9529 (30.7)	12 569 (31.3)
Postintervention (2011‐12)	96 (39.2)	3073 (39.1)	11 511 (40.5)	14 680 (40.0)
Age				
50‐54 y	105 (42.3)	1305 (22.8)	6772 (28.9)	8182 (27.8)
55‐59 y	72 (25.6)	1817 (25.2)	7053 (25.5)	8942 (25.4)
60‐64 y	51 (14.2)	2028 (22.3)	6402 (19.7)	8481 (20.4)
65‐69 y	43 (11.5)	1883 (17.4)	5050 (14.2)	6976 (15.2)
70‐74 y	25 (6.5)	1544 (12.4)	4149 (10.7)	5718 (11.2)
Educational attainment				
Less than secondary school graduation	45 (19.0)	1548 (17.7)	6074 (17.1)	7667 (17.4)
Secondary school graduation	51 (12.7)	1534 (17.1)	5719 (19.5)	7304 (18.5)
Some postsecondary school education	7 (2.0)	356 (4.5)	1537 (5.8)	1900 (5.2)
Postsecondary school graduation	193 (66.4)	5139 (60.7)	16 096 (57.6)	21 428 (58.9)
Household income				
No income or <$20 000	61 (16.0)	861 (7.1)	3005 (6.3)	3927 (6.9)
$20 000‐$39 999	68 (14.6)	2019 (17.6)	6144 (14.9)	8231 (15.8)
$40 000‐$59 999	54 (19.2)	1922 (21.6)	6514 (19.1)	8490 (20.0)
$60 000‐$79 999	43 (22.3)	1497 (19.3)	5471 (19.0)	7011 (18.8)
$80 000 or more	70 (28.0)	2278 (35.5)	8292 (20.6)	10 640 (38.5)
Self‐perceived health				
Excellent	46 (10.7)	1474 (16.5)	5167 (19.3)	6687 (18.1)
Very good	96 (34.3)	2836 (33.1)	10 782 (38.1)	13 714 (36.3)
Good	111 (38.4)	2808 (34.1)	8541 (28.2)	11 460 (30.4)
Fair	30 (10.2)	1041 (11.6)	3566 (10.3)	4637 (10.8)
Poor	13 (6.4)	418 (4.1)	1370 (4.1)	1801 (4.4)

All percentages are weighted.

Across the 3 time periods, 28.8% of recent immigrants reported ever being screened for CRC using either FOBT or sigmoidoscopy screening methods, while 58.6% and 63.7% of long‐term immigrants and Canadian‐born individuals, respectively, reported ever having been screened (Table 1). In regard to screening adherence within the *ColonCancerCheck* recommended time interval, the proportion dropped to 22.6% of recent immigrants reporting having been screened using FOBT within the last 2 years or endoscopy within the last 10 years, while 50.3% of long‐term immigrants and 53.9% of Canadian‐born individuals had adhered to CRC screening guidelines.

The overall sample was evenly distributed across males and females, although there was a slight over‐representation of males among recent immigrants (54.3% male vs. 45.7% female). The sample was fairly evenly distributed across the 5 age groups, with the highest proportion of individuals being in the 50‐54 years age group (27.8%, n = 8182). A majority of participants (58.9%, n = 21 428) reported the highest possible level of educational attainment, “Post‐Secondary Graduation”, and more than 80% (n = 31 861) of the overall sample reported “Good” or better self‐perceived health. Amongst the overall sample, 38.5% (n = 10 640) reported being in the highest income bracket (household annual income of $80 000 or more), while 6.9% were in the lowest income bracket (no income or <$20 000). Of note, recent immigrants had a higher proportion (16.0%, n = 61) of individuals in the lowest income bracket than long‐term immigrants (7.1%, n = 861) or Canadian‐born individuals (6.3%, n = 3005).

### 3.2 Association between colon cancer screening adherence and immigration status

Table 2 shows adjusted odds ratios for the association between immigration status and cancer screening nonadherence, adjusted for the influence of sex, age, educational attainment, household income, and self‐perceived health. In this model, recent immigrants were shown to have more than 3 times the odds of CRC screening nonadherence when compared to Canadian‐born individuals (OR 3.73 [CI 2.25‐6.18]). Long‐term immigrants also had a statistically significant higher, though attenuated, odds of nonadherence compared to Canadian‐born individuals (OR 1.24 [CI 1.13‐1.36]).

**Table 2:** Adjusted odds ratios and 95% confidence intervals for the effect on colorectal cancer screening nonadherence

Screening nonadherence Main effects model	AOR (95% CI)	*p*‐value for variable trend
Immigration status		
Canadian‐born individuals	1.00	<0.01
Long‐term immigrants	1.24 (1.13‐1.36)	
Recent immigrants	3.73 (2.25‐6.18)	
Intervention period		
Postintervention (2011‐12)	1.00	<0.01
Roll‐out (2007‐08)	1.65 (1.50‐1.82)	
Preintervention (2005)	2.47 (2.24‐2.72)	
Sex		
Female	1.00	<0.01
Male	1.16 (1.07‐1.25)	
Age		
70‐74	1.00	<0.01
65‐69	1.03 (0.91‐1.17)	
60‐64	1.20 (1.06‐1.37)	
55‐59	1.68 (1.48‐1.91)	
50‐54	2.84 (2.48‐3.25)	
Educational attainment		
Postsecondary school graduation	1.00	<0.01
Some postsecondary school education	0.88 (0.73‐1.06)	
Secondary school graduation	0.86 (0.77‐0.95)	
Less than secondary school graduation	1.21 (1.06‐1.37)	
Self‐perceived health		
Poor	1.00	0.01
Fair	1.11 (0.89‐1.39)	
Good	1.15 (0.94‐1.42)	
Very good	1.28 (1.04‐1.57)	
Excellent	1.33 (1.07‐1.65)	
Household Income		
$80 000 or more	1.00	<0.01
$60 000‐$79 999	1.34 (1.20‐1.51)	
$40 000‐$59 999	1.34 (1.19‐1.50)	
$20 000‐$39 999	1.60 (1.41‐1.81)	
<$20 000	1.90 (1.62‐2.23)	

All statistics are weighted.

AOR, Adjusted odds ratio; CI, confidence interval.

The adjusted effects of confounders showed trends consistent with past studies examining the association between CRC screening uptake and immigration status (Table 2). All covariates included in the model were statistically significant predictors of cancer screening. Most notably, the odds of nonadherence in the preintervention period was over 2 times that of the postintervention period (OR 2.47 [CI 2.24‐2.72]), and the odds of nonadherence in the roll‐out period was 65% higher than in the postintervention period (OR 1.65 [CI 1.50‐1.82]). Nonadherence was also statistically significantly higher for males than for females (OR 1.16 [CI 1.07‐1.25]), and also appears to decrease with increasing age, household income, and educational attainment.

The effect of immigration status on CRC screening adherence did not significantly differ between the 3 intervention periods (all *P* > 0.1); however, the effect of immigration status on colon screening was attenuated after introduction of the *ColonCancerCheck* program (Table 3). In all time periods, the odds of nonadherence to colorectal cancer screening was higher for immigrants than for Canadian‐born individuals. The odds of nonadherence for recent immigrants was over 4 times that of Canadian‐born individuals in the preintervention period (AOR 5.60 [CI 2.71‐11.58]), attenuating to almost 4 times the odds in the roll‐out period (AOR 3.69 [CI 1.94‐7.03]), and to just over 3 times the odds in the postintervention period (AOR 3.09 [CI 1.27‐7.55]).

**Table 3:** Adjusted odds ratios and 95% confidence intervals association between immigration status and time‐appropriate colorectal cancer screening, by *ColonCancerCheck *intervention period

Immigration status	Preintervention (2005’)	Roll‐out (2007‐08)	Postintervention (2011‐12)
	Adjusted^a^ OR (95% CI)	Adjusted^a^ OR (95% CI)	Adjusted^a^ OR (95% CI)
Recent immigrants	5.60 (2.71‐11.58)	3.69 (1.94‐7.03)	3.09 (1.27‐7.55)
Long‐term immigrants	1.15 (1.00‐1.33)	1.37 (1.81‐1.58)	1.17 (0.98‐1.39)
Canadian‐born	1.00	1.00	1.00

All Statistics are weighted

AOR, Adjusted odds ratio; CI, confidence interval

a Model adjusted for all previously listed covariates – sex, age category, educational attainment, self‐perceived health, and household income

Among long‐term immigrants, the odds of nonadherence were 15% higher compared with Canadian‐born individuals in the preintervention period (AOR 1.15 [CI 1.00‐1.33]), which increased to 37% in the roll‐out period (AOR 1.37 [CI 1.81‐1.58]), and decreased again to 17% higher odds compared to Canadian‐born in the postintervention period (OR 1.17 [CI 0.98‐1.39]).

## 4 DISCUSSION

This study first sought to determine the association between immigration status and CRC screening nonadherence (screening using FOBT within the last 2 years or using sigmoidoscopy within the last 10 years). The association was examined for 3 distinct groups: recent immigrants (those having arrived in Canada between 0 and 9 years before the survey date), long‐term immigrants (those having arrived in Canada 10 or more years before the survey date), and Canadian‐born individuals (nonimmigrants). Furthermore, this study sought to determine the effect of the implementation of the *ColonCancerCheck* population screening program, characterized by the time periods prior to, during the roll‐out of, and after complete implementation of the *ColonCancerCheck* intervention, by examining the interacting effect of immigration status and intervention period on CRC screening nonadherence.

After controlling for confounders, we found evidence of higher odds of screening nonadherence for recent and long‐term immigrants as compared to Canadian‐born individuals (Table 2). Furthermore, while the interaction terms between immigration status and intervention period were not statistically significant, the interaction analysis suggests that screening nonadherence decreased with the implementation of the *ColonCancerCheck *program for recent immigrants compared with Canadian‐born individuals (Table 3), although the odds of nonadherence remained higher for both recent and long‐term immigrants compared with Canadian‐born.

Our findings are consistent with an earlier study by Honein‐AbouHaidar describing differences in CRC screening participation in Ontario between immigrant and nonimmigrant populations 3 years before and 3 years after the *ColonCancerCheck* program was introduced. This study found that between 2005 and 2011, overall screening rates in Ontario rose steadily, however the immigrant population was consistently and significantly less likely to be up‐to‐date with CRC screening compared to nonimmigrants before and after the introduction of the population screening program.^5^ Results from Kiran et al’s 2017 paper, where CRC screening rates for eligible Ontarians in each year between 2001 and 2014 were calculated and immigrant‐related screening disparities were quantified using an adjusted ratio of screening rates, also resulted in modest decreases in disparities, represented by an increase in the screening rate ratio from 0.55 to 0.69.^4^

Our main findings are also consistent with past studies showing a differential between immigrant CRC screening adherence, compared to the Canadian‐born population. We hypothesized that programmatic CRC screening would help reduce screening nonadherence for immigrant and nonimmigrant populations. While not statistically significant, the difference between recent immigrant and nonimmigrant colon screening adherence did reduce with the introduction of the *ColonCancerCheck *program. Evidence suggests that immigrant populations face a number of barriers to health care access, notably due to a lack of information or knowledge of Canada's health care system and screening practices,^6^ as well as due to increased language and cultural barriers.^27^ The *ColonCancerCheck* and other formal population screening programs do provide explicit and targeted population‐wide coverage, which may mitigate accessibility barriers to participation among minority populations, especially those related to a lack of information and knowledge. Is it also possible that recent immigrants may suffer from higher rates of nonadherence due to the lack of colorectal cancer screening knowledge or availability in their country of origin.

This study has a number of strengths and limitations. The representativeness of the CCHS survey sample makes results generalizable to Ontario's wider population. The use of repeated cross‐sectional study data is also beneficial compared with cohort designs, in that it does not suffer from losses of follow‐up.^28^ While the aforementioned studies offered important preliminary evidence of the association between the implementation of the *ColonCancerCheck* program, an uptake of immigrants, their use of administrative data and ecological level proxy measures for immigration status and income may have resulted in important differential misclassification and bias toward the null.^4^, ^5^ The literature also suggests that the use of area‐based estimates of household income demonstrates poor agreement with individual or self‐reported household income, particularly for low‐income groups.^29^, ^30^, ^31^ This study is the first to use survey data with self‐identified immigration status, while controlling for the effect of known confounders. Data included in this study on educational attainment is thought to be especially valuable given the heterogeneity of the immigrant population – for example, economic class immigrants (skilled workers, business immigrants) are specifically accepted into Canada for their high educational achievement and are likely to face substantially different barriers to screening compared to family class immigrants or refugees.

Our evaluation of the *ColonCancerCheck* program relied on cross‐sectional data to capture the effects of a natural experiment. As such, it was not possible to isolate the effect of the screening program, or to measure the effect of temporal trends. Data limitations also prevented us from comparing Ontario's immigrant population to that of other provinces where no population colorectal screening program exists. More extensive data are required to perform similar analyses in other provinces.

The CCHS is based on self‐reported data, and our study is thus vulnerable to recall and social desirability bias, including telescoping bias. Telescoping bias is described in the literature as participants recalling events occurring more recently than they actually did.^32^ Some evidence suggests that immigrants are more susceptible to such over‐estimation,^31^ which may have biased results toward the null. It is also possible that individuals will have reported better screening adherence in order to be viewed favorably by the interviewer, known as social desirability bias. In addition, the CCHS survey is generally conducted in English or French, with limited translation available depending on language competencies of individuals within the participant's household or among interviewers. Limited translation services may have restricted the participation of recent immigrants who are not yet fluent in the official languages, and would have biased results toward the null^19^. Lastly, a noteworthy number of observations (n = 4899, or 1.3% of the total sample) were excluded due to nonresponse or invalid responses to household income, educational attainment, and self‐perceived health. If these went unreported due to the sensitive nature of these topics or due to the lack of knowledge of colorectal cancer, it is possible that there was a loss of some vulnerable individuals in the study and an attenuation of the estimated odds of nonadherence.

Our study adds important data to the limited literature examining the effects of population CRC screening programs in Canada. Our analysis provides evidence that the *ColonCancerCheck* program in Ontario helped attenuate the odds of CRC screening nonadherence in immigrant and nonimmigrant populations. These findings provide additional justification that organized screening programs lead to less disparity than ad hoc screening, and may therefore help inform necessary implementation of additional screening programs, such as lung cancer screening. Future studies should examine predictive factors for colon cancer screening among immigrants in order to develop targeted interventions that address barriers to preventative health services in underserved populations.

## CONFLICT OF INTEREST

None to declare.
